# Cancer incidence and mortality in China, 2016^☆^

**DOI:** 10.1016/j.jncc.2022.02.002

**Published:** 2022-02-27

**Authors:** Rongshou Zheng, Siwei Zhang, Hongmei Zeng, Shaoming Wang, Kexin Sun, Ru Chen, Li Li, Wenqiang Wei, Jie He

**Affiliations:** 1 Office for Cancer Registry, National Cancer Center/National Clinical Research Center for Cancer/Cancer Hospital, Chinese Academy of Medical Sciences and Peking Union Medical College, Beijing 100021, China; 2 Department of Thoracic Surgery, National Cancer Center/National Clinical Research Center for Cancer/Cancer Hospital, Chinese Academy of Medical Sciences and Peking Union Medical College, Beijing 100021, China

**Keywords:** Cancer registry, Incidence, Mortality, Statistics, China

## Abstract

**Background:**

National Cancer Center (NCC) of China annually reports the nationwide statistics for cancer incidence and mortality using population-based cancer registry data from all available cancer registries in China.

**Methods:**

There were a total of 487 registries which reported high quality data of cancer incidence and mortality across China in 2016. The nationwide numbers of new cancer cases and deaths were estimated using the pooled cancer registry data, which were stratified by area (urban/rural), sex, age group (0, 1-4, 5-9, 10-14…85+) and cancer site for incidence and mortality, and then multiplied by corresponding national population. The world Segi's population was applied for the calculation of age-standardized rates.

**Results:**

About 4,064,000 new cancer cases and 2,413,500 new cancer deaths occurred in China in 2016. Cancers of the lung, colon-rectum, stomach, liver and female breast were the top five common cancers, accounting for 57.4% of total cancer new cases. Cancers of the lung, liver, stomach, colon-rectum and esophagus were the five leading causes of cancer deaths, accounting for 69.3% of total cancer deaths. The crude and age-standardized incidence rates (ASIR) were 293.91 and 186.46 per 100,000 population, respectively. The crude mortality rate was 174.55/100,000 and the age-standardized mortality rate (ASMR) was 105.19/100,000. The ASIR was higher but the ASMR was lower in urban areas than that in rural areas. In past decades, the ASIR was relatively stable in males, but significantly increased by about 2.3% per year in females for overall cancers combined. In contrast, the ASMR significantly decreased by about 1.2% per year for both sexes during 2000-2016. Notably, the cancer-specific ASIR and ASMR of esophageal, stomach, and liver cancers decreased significantly, whereas both rates for cancers of the colon-rectum, prostate, female breast, cervix, and thyroid increased significantly.

**Conclusions:**

Cancer remains a major public health problem in China, which demands long-term collaborative efforts of a broad community. With the national guideline on cancer prevention and control, tailored cancer prevention and control programs are needed in different regions to help reduce the burden of these highly fatal diseases in China.

## 1 Introduction

Cancer is a major public health problem and has become one of the most common causes of death in China^1^. Cancer registries can be used for continuous and dynamic monitoring of cancer incidence and mortality. Cancer Registration is the fundamental work for formulating cancer prevention and control strategies, launching comprehensive prevention and control research, and evaluating effects on prevention and control^2^. China has established a nationwide cancer registration and follow-up surveillance system, which can continuously release Cancer Registry Annual Report. By the end of 2020, cancer registration had covered 1152 counties with a population coverage of 598 million. National Cancer Center (NCC) is responsible for collecting, evaluating, and publishing the national cancer statistics of China. All hospitals and medical and health institutions in the administrative regions are required to submit cancer records to local population-based cancer registries.

This report provided the latest statistics of new cancer incidence and mortality in China in 2016, and comprehensively estimated the overall numbers of new cancer cases and deaths in 2016. We further updated the trends of cancer incidence and mortality from 2000 to 2016. This up-to-date nationwide cancer profiles can provide scientific evidence for cancer prevention and control in China.

## 2 Materials and methods

### 2.1 Quality control

NCC is responsible for data quality control, including assessing the validity, reliability, completeness and comparability of all cancer registry data based on the criteria of “Guideline for Chinese Cancer Registration”^3^ and criteria of International Agency for Research on Cancer/International Association of Cancer Registries (IARC/IACR)^4^^,^^5^. Indexes including mortality to incidence (M/I) ratio, proportion of cases with morphological verification (MV), percentage of cases with death-certificate-only (DCO), percentage of cancer diagnosis with unknown basis (UB) and the stability of cancer trends over years were used for quality control.

### 2.2 Data source

By 31^th^ December 2019, a total of 682 cancer registries from 31 provinces (autonomous regions and municipalities) and Xinjiang Production and Construction Corps (not including Hong Kong, Macao Special Administrative Regions and Taiwan Province) submitted registration data of 2016 to National Cancer Center (NCC). All newly diagnosed cancer cases were coded according to the International Classification of Diseases for Oncology, 3^rd^ edition (ICD-O-3) and the International Statistical Classification of Diseases 10^th^ Revision (ICD-10).

Temporal trends for age-standardized rates by world standard population from 2000 to 2016 for cancer incidence and mortality of all cancers and selected cancer types were analyzed using data from 22 continuous cancer registries, which represented 3.34% of the Chinese population. More details about those registries have been reported in a previous study^1^.

The National Bureau of Statistics of China provided the total number of population of China in 2016, stratified by area (urban/rural) and sex. The age-specific population of 2016 was estimated according to the population structure of the data of the fifth and sixth National Census, which provided the data of the whole population in group (0-, 1-4, 5-84 by 5 years and 85+ years).

### 2.3 Statistical analysis

Cancer incidence and mortality rates stratified by age (0-, 1-4, 5-84 by 5 years and 85+ years), sex (male/female), area (urban/rural) and region (seven administrative regions including North, Northeast, East, Central, South, Southwest, and Northwest) were calculated using pooled qualified cancer registries’ data. The incidence and mortality rates were multiplied with the population in each strata and then summed up to obtain the estimated numbers of new cancer cases and deaths. The Segi's population was used for age-standardized rates. If a registry is located in a county, it was classified as a rural registry, while it was classified as an urban registry if it is located in a city. The classification of seven administrative regions was based on that of the National Bureau of Statistics. All models were restricted to a maximum of 2 joinpoints (3 line segments). The annual percent change (APC) and the average Annual Percent Change (AAPC) for three fixed intervals (2000-2016, 2007-2016 and 2011-2016) were calculated using Joinpoint Regression Program (version 4.6.0.0) for both incidence and mortality. SAS software (Version 9.4, SAS Institute Inc., Cary, USA) was used for statistical analysis.

## 3 Results

After data quality control, 487 cancer registries’ data were qualified and included in this analysis, of which 200 registries were from rural areas and 287 were from urban areas. The population covered by these cancer registries was 381,565,422 (193,632,323 males and 187,933,099 females), accounting for 27.60% (24.3% for urban areas and 32.0% for rural areas) of the national population at the end of 2016.

### 3.1 Estimated numbers of new cancer cases and cancer incidence rates

Table 1 shows the estimated numbers of new cancer cases and deaths in China in 2016. Overall, an estimated number of 4,064,000 new cancer cases occurred in 2016. Lung cancer was the most common cancer in men, accounting for about 24.6% (549,800) of all new cancer cases, followed by liver, stomach, colorectal and esophageal cancers. These top five cancers accounted for about 68.83 % of all newly diagnosed cancers in men. In women, breast cancer was the most common, accounting for 16.72% (306,000) of all new cancer cases, followed by lung, colorectal, thyroid and stomach cancers. These top five cancers accounted for about 56.11% of all new cancer diagnosis in women.

**Table 1:** Estimated numbers of new cancer cases and incidence rates, overall, by sex and cancer type in China, 2016.

ICD10	Site	All			Men			Women		
		Cases	Crude incidence (1/10^5^)	ASIRW (1/10^5^)	Cases	Crude incidence (1/10^5^)	ASIRW (1/10^5^)	Cases	Crude incidence (1/10^5^)	ASIRW (1/10^5^)
C00-10, 12-14	Lip, oral cavity & pharynx	52200	3.78	2.43	36200	5.11	3.38	16100	2.38	1.48
C11	Nasopharynx	52000	3.76	2.51	37400	5.28	3.58	14700	2.17	1.42
C15	Esophagus	252500	18.26	11.13	184500	26.05	16.81	68000	10.07	5.60
C16	Stomach	396500	28.68	17.59	276300	39.02	25.14	120200	17.82	10.31
C18-21	Colorectum	408000	29.51	18.05	238500	33.68	21.65	169500	25.13	14.58
C22	Liver	388800	28.12	17.65	288800	40.78	26.65	100000	14.83	8.65
C23, 24	Gallbladder	55700	4.03	2.39	26400	3.73	2.36	29300	4.34	2.41
C25	Pancreas	100400	7.26	4.36	57000	8.05	5.14	43400	6.43	3.60
C32	Larynx	25700	1.86	1.17	23500	3.32	2.16	2200	0.33	0.19
C33, 34	Lung	828100	59.89	36.46	549800	77.64	49.78	278300	41.26	23.70
C37, 38	Other thoracic organs	13100	0.95	0.64	7600	1.08	0.75	5500	0.81	0.54
C40, 41	Bone	25800	1.87	1.37	14900	2.11	1.59	10900	1.62	1.16
C43	Melanoma of skin	7000	0.51	0.32	3500	0.50	0.32	3500	0.52	0.32
C50	Female breast	306000	45.37	29.05	—	—	—	306000	45.37	29.05
C53	Cervix	119300	17.69	11.34	—	—	—	119300	17.69	11.34
C54, 55	Uterus	71100	10.54	6.64	—	—	—	71100	10.54	6.64
C56	Ovary	57200	8.47	5.59	—	—	—	57200	8.47	5.59
C61	Prostate	78300	11.05	6.72	78300	11.05	6.72	—	—	—
C62	Testis	3400	0.48	0.41	3400	0.48	0.41	—	—	—
C64-66, 68	Kidney	75800	5.48	3.51	48000	6.78	4.51	27800	4.12	2.53
C67	Bladder	82300	5.95	3.53	64200	9.07	5.71	18000	2.67	1.49
C70-72	Brain, CNS	109000	7.88	5.57	50500	7.13	5.26	58500	8.67	5.87
C73	Thyroid	202600	14.65	10.37	50000	7.06	5.11	152600	22.63	15.81
C81-85, 88, 90, 96	Lymphoma	89900	6.50	4.36	51600	7.29	5.07	38300	5.67	3.67
C91-95	Leukemia	85800	6.21	5.10	49400	6.97	5.77	36400	5.40	4.42
Other	All other sites	173600	12.55	8.28	90900	12.83	8.81	82700	12.26	7.78
ALL	All sites	4064000	293.91	186.46	2234300	315.52	207.03	1829600	271.23	168.14

Abbreviations: ASIRW, age-standardized incidence rate by world standard population (Segi's population); CNS, central nervous system; ICD-10, International Statistical Classification of Diseases 10^th^ Revision.

Table 2 displays the ASIRs of all cancers combined and 12 selected cancer sites in China in 2016. The age-standardized incidence rate (ASIR) for all cancers combined in urban areas was higher than that in rural areas (189.7/100,000 vs. 176.2/100,000). South China had the highest ASIR (204.3/100,000), followed by Northeast China and East China. Southwest China (167.5/100,000) had the lowest ASIR. The incidence rates of cancers of colon-rectum, lung, female breast and prostate in urban areas were higher than the rates of those cancers in rural areas. However, the ASIR for some digestive cancers including esophageal cancer, gastric cancer, and liver cancer were lower in urban areas than that in rural areas.

**Table 2:** Age-standardized incidence rates overall, by area and cancer type by geographic areas covered by 487 cancer registries, 2016 (1/10^5^).

Geographic areas	All sites	Esophagus	Stomach	Colorectum	Liver	Lung	Female breast	Cervix	Prostate	Kidney	Bladder	Lymphoma	Leukemia
All areas	183.3	11.5	17.6	17.4	17.7	36.0	28.0	11.4	6.4	3.3	3.4	4.2	5.0
Urban	189.7	8.2	15.5	20.0	16.3	36.7	31.8	10.9	8.2	4.1	3.8	4.8	5.1
Rural	176.2	15.0	19.8	14.7	19.3	35.2	23.8	11.9	4.4	2.5	3.0	3.6	4.9
North	180.1	8.2	14.6	16.3	12.5	35.0	34.0	8.4	6.1	5.5	4.4	4.6	5.9
Northeast	188.4	4.6	13.8	21.1	18.6	41.6	34.1	11.6	4.7	4.8	4.9	3.0	3.7
East	186.5	13.5	21.9	17.3	16.6	35.3	27.7	10.7	7.6	3.2	3.2	4.3	5.0
Central	183.2	14.3	18.7	15.3	18.5	36.5	28.0	14.9	4.5	2.4	2.9	4.3	5.3
South	204.3	5.1	9.2	23.8	26.1	34.9	34.8	12.1	8.6	2.9	3.3	5.9	6.4
Southwest	167.5	13.5	13.1	16.5	19.2	38.6	18.6	11.9	5.1	1.9	2.9	3.5	4.3
Northwest	175.2	12.1	25.8	14.5	18.1	28.9	21.6	12.7	5.6	3.5	3.8	3.1	3.9
North (Urban)	186.5	4.3	11.5	18.8	10.5	34.0	40.0	7.7	8.2	6.8	5.1	5.4	6.5
Northeast (Urban)	196.9	4.1	13.6	23.1	15.9	42.6	38.3	12.6	5.6	5.6	5.2	3.4	3.8
East (Urban)	196.3	9.7	20.6	20.2	15.4	36.0	31.5	10.2	10.1	4.0	3.5	4.9	5.1
Central (Urban)	184.1	9.8	14.3	17.9	15.9	37.2	31.3	12.8	6.5	3.1	3.5	4.9	5.3
South (Urban)	212.3	4.6	8.6	26.0	24.1	35.9	38.7	11.4	10.2	3.2	3.7	6.5	6.6
Southwest (Urban)	169.4	10.6	10.3	18.0	18.0	40.8	21.0	12.0	6.4	2.3	3.1	4.0	4.2
Northwest (Urban)	176.5	11.6	25.0	15.0	17.3	29.2	22.6	12.4	6.2	3.9	3.8	3.5	4.2
North (Rural)	173.9	12.3	17.8	13.5	14.6	36.0	28.1	9.0	3.9	4.2	3.7	3.7	5.4
Northeast (Rural)	169.9	5.7	14.1	16.2	24.8	39.3	25.0	9.3	2.8	3.1	4.4	2.2	3.4
East (Rural)	177.5	16.9	23.1	14.6	17.7	34.6	24.1	11.1	5.2	2.4	2.9	3.8	4.9
Central (Rural)	182.6	17.4	21.8	13.4	20.3	36.0	25.7	16.2	3.2	2.0	2.5	3.8	5.3
South (Rural)	190.5	5.9	10.3	19.8	30.0	33.1	27.4	13.4	5.8	2.3	2.7	4.9	6.0
Southwest (Rural)	165.7	16.4	15.8	15.1	20.4	36.4	16.2	11.9	3.9	1.5	2.7	3.1	4.4
Northwest (Rural)	170.2	13.9	28.5	12.7	20.9	27.7	17.6	13.8	3.4	2.3	3.5	1.9	2.7

### 3.2 Estimated numbers of cancer deaths and cancer mortality rates

The estimated numbers of total deaths for all cancers and 26 cancer types stratified by sex were shown in Table 3. About 2,413,500 people died from cancer in China in 2016. Lung cancer was the most common cause of cancer death for both sexes. For men, lung cancer deaths accounted for about 29.71% (454,700) of all cancer deaths, followed by liver cancer, stomach cancer, esophageal cancer and colorectal cancer. The five leading causes of cancer deaths accounted for about 75.87% of all cancer deaths in males. For women, lung cancer was the most common cause of cancer deaths, followed by stomach cancer, liver cancer, colorectal cancer, and breast cancer. These five leading causes of cancer death accounted for about 60.06% of all cancer deaths in women.

**Table 3:** Estimated cancer deaths and mortality rates, overall, by sex and cancer type in China, 2016.

ICD10	Site	All			Male			Female		
		Deaths	Crude mortality (1/10^5^)	ASMRW (1/10^5^)	Deaths	Crude mortality (1/10^5^)	ASMRW (1/10^5^)	Deaths	Crude mortality (1/10^5^)	ASMRW (1/10^5^)
C00-10,12-14	Lip, oral cavity & pharynx	25800	1.87	1.13	18900	2.67	1.71	6900	1.03	0.56
C11	Nasopharynx	26700	1.93	1.24	19700	2.78	1.84	7000	1.04	0.63
C15	Esophagus	193900	14.02	8.28	142300	20.10	12.73	51600	7.64	4.00
C16	Stomach	288500	20.87	12.30	200200	28.27	17.77	88400	13.10	7.13
C18-21	Colon-rectum	195600	14.14	8.13	114500	16.17	10.04	81000	12.01	6.36
C22	Liver	336400	24.33	15.07	249600	35.25	22.90	86800	12.86	7.27
C23-24	Gallbladder	41400	3.00	1.73	19500	2.75	1.71	22000	3.25	1.73
C25	Pancreas	87900	6.35	3.75	49800	7.03	4.44	38100	5.64	3.08
C32	Larynx	14300	1.03	0.61	12500	1.77	1.12	1700	0.26	0.14
C33-34	Lung	657000	47.51	28.09	454700	64.21	40.58	202300	29.99	16.24
C37-38	Other thoracic organs	6800	0.49	0.32	4300	0.61	0.41	2500	0.37	0.23
C40-41	Bone	18400	1.33	0.88	10700	1.52	1.06	7700	1.14	0.71
C43	Melanoma of skin	3800	0.28	0.17	2100	0.30	0.20	1700	0.25	0.14
C50	Female breast	71700	10.62	6.39	—	—	—	71700	10.62	6.39
C53	Cervix	37200	5.52	3.36	—	—	—	37200	5.52	3.36
C54-55	Uterus	17100	2.53	1.51	—	—	—	17100	2.53	1.51
C56	Ovary	27200	4.04	2.45	—	—	—	27200	4.04	2.45
C61	Prostate	33600	4.75	2.73	33600	4.75	2.73	—	—	—
C62	Testis	900	0.12	0.09	900	0.12	0.09	—	—	—
C64-66, 68	Kidney	26900	1.95	1.17	17100	2.42	1.55	9800	1.45	0.81
C67	Bladder	33700	2.44	1.31	26200	3.69	2.20	7500	1.12	0.54
C70-72	Brain, CNS	58500	4.23	2.91	32600	4.61	3.31	25900	3.83	2.51
C73	Thyroid	8300	0.60	0.37	3100	0.44	0.29	5200	0.77	0.45
C81-85,88,90,96	Lymphoma	51500	3.73	2.34	31000	4.38	2.91	20500	3.03	1.80
C91-95	Leukemia	55700	4.03	2.98	32400	4.58	3.49	23200	3.45	2.48
A_O	All other sites	93400	6.76	4.18	53500	7.56	4.97	39900	5.92	3.41
ALL	All sites	2413500	174.55	105.19	1530700	216.16	138.14	882800	130.88	73.95

Abbreviations: ASMRW, age-standardized mortality rate by world standard population (Segi's population); CNS, central nervous system; ICD-10, International Statistical Classification of Diseases and Related Health Problems 10^th^ Revision.

Table 4 showed the age-standardized mortality rates (ASMR) for all cancers and 12 selected cancers in China in 2016. The ASMR for all cancers in rural areas was higher than in urban areas (106.1/100,000 vs. 102.8/100,000). Central China had the highest cancer mortality rate (112.0/100,000), followed by Northeast China and South China. North China (94.5/100,000) had the lowest mortality rate. The patterns of cancer mortality was observed to be varied in different regions. Mortality rates in urban areas for cancers of colon-rectum, lung, female breast, prostate, kidney, bladder, lymphoma and leukemia were higher than those in rural areas, whereas higher rates were observed in rural areas for digestive cancers such as esophageal cancer, gastric cancer, and liver cancer than those in urban areas.

**Table 4:** Age-standardized mortality rates for cancer, overall, by area and cancer type by geographic areas covered by 487 cancer registries, 2016 (1/10^5^).

Geographic areas	All sites	Esophagus	Stomach	Colorectum	Liver	Lung	Female breast	Cervix	Prostate	Kidney	Bladder	Lymphoma	Leukemia
All areas	104.5	8.5	12.4	7.9	15.2	27.9	6.2	3.4	2.6	1.1	1.3	2.3	3.0
Urban	102.8	6.2	10.6	9.0	13.9	28.0	7.0	3.3	3.1	1.4	1.4	2.5	3.0
Rural	106.1	11.0	14.3	6.7	16.6	27.6	5.4	3.5	2.0	0.8	1.1	2.1	2.9
North	94.5	5.6	10.3	7.3	10.5	27.1	6.4	2.5	2.6	1.6	1.5	2.5	3.2
Northeast	108.2	3.9	9.5	10.0	15.7	32.5	7.5	3.3	2.7	1.9	2.0	1.6	2.1
East	104.0	10.1	14.8	7.5	14.3	26.1	5.6	2.7	2.7	1.0	1.2	2.5	2.9
Central	112.0	10.5	13.8	7.6	15.8	30.1	7.5	5.0	2.3	1.0	1.2	2.4	3.3
South	108.1	4.1	6.4	9.8	22.3	27.7	7.6	3.6	3.2	1.0	1.1	2.8	3.4
Southwest	103.6	9.7	9.9	7.9	17.1	30.2	4.8	3.9	2.1	0.6	1.1	1.8	2.8
Northwest	106.1	8.3	18.1	7.3	14.5	23.3	6.7	4.4	3.0	1.4	1.3	1.5	2.4
North (Urban)	90.0	3.5	7.7	8.2	9.0	25.5	7.7	2.3	3.1	2.0	1.6	3.0	3.2
Northeast (Urban)	109.7	3.6	9.5	11.1	13.4	32.9	8.6	3.7	3.1	2.1	2.0	1.8	2.0
East (Urban)	101.5	7.5	13.6	8.7	13.1	25.5	6.2	2.5	3.3	1.2	1.3	2.6	3.0
Central (Urban)	107.6	7.4	10.1	8.8	13.7	30.9	8.1	4.4	3.1	1.3	1.4	2.8	3.3
South (Urban)	106.6	3.7	5.7	10.7	20.1	28.1	8.0	3.2	3.8	1.0	1.2	3.1	3.4
Southwest (Urban)	103.8	7.7	7.7	8.6	16.2	32.8	5.3	4.1	2.5	0.8	1.2	2.1	2.9
Northwest (Urban)	107.8	8.1	17.7	7.5	13.9	24.2	7.1	4.5	3.2	1.6	1.2	1.6	2.6
North (Rural)	98.7	8.0	13.2	6.1	12.0	28.6	4.9	2.8	1.8	1.1	1.4	2.0	3.0
Northeast (Rural)	104.2	4.6	9.6	7.3	21.0	31.5	5.1	2.5	1.7	1.3	1.8	1.1	2.3
East (Rural)	106.1	12.6	15.9	6.3	15.4	26.7	5.1	2.9	2.2	0.8	1.0	2.3	2.9
Central (Rural)	114.8	12.7	16.4	6.7	17.3	29.4	7.2	5.4	1.7	0.8	1.1	2.2	3.3
South (Rural)	111.6	4.9	7.6	8.4	26.6	27.2	6.8	4.4	2.3	1.0	1.0	2.5	3.4
Southwest (Rural)	103.4	11.7	12.1	7.2	17.9	27.6	4.3	3.8	1.7	0.5	1.0	1.6	2.7
Northwest (Rural)	99.8	9.1	20.0	6.2	16.3	19.7	5.2	4.1	2.1	0.7	1.3	1.0	1.7

### 3.3 Age-specific incidence and mortality

**Fig. 1:**
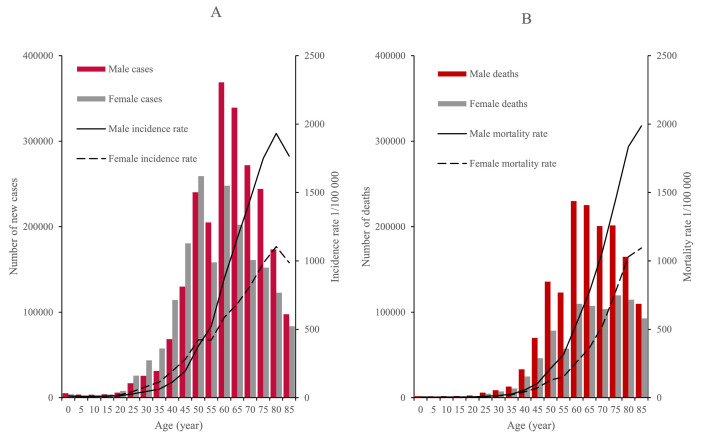
Age-specific cancer incidence and mortality by sex in China, 2016. (A) Age-specific cancer incidence rates and number of new cases by sex. (B) Age-specific cancer mortality rates and deaths by sex.

Fig. 1 showed that cancer incidence and mortality rates both increased with age, and reached the peak in the age groups of 80-84 years and 85+ years for both sexes, respectively. The age groups of 60-64 and 50-54 years were subjected to most cancer cases, and the age groups of 60-64 and 75-79 years accrued most cancer deaths in males and females, respectively. In general, males had higher incidence and mortality rates than females, except that the incidence rate of females in the group aged 20-49 was higher than that of males. (Fig. 1A and B).

### 3.4 Estimated numbers of new cancer cases and deaths by age groups and sex

Table 5 shows the estimated new cancer cases of the top five cancers types and all cancers by age group and sex. The age group of 60-79 years accrued the most cancer cases, which were 1.22 million and 763,000 in males and females, respectively. Leukemia, brain cancer and lymphoma were ranked as the three cancer types with the highest incidences in both sexes. Lung cancer was the most common cancer in men for the population aged at 45 and above. Breast cancer and lung cancer were the most common cancers in women in the age groups of 15-59 years and 60+ years, respectively.

**Table 5:** Estimated numbers of new cancer cases of all cancers and five leading cancer types by age and sex in China, 2016 (thousands).

Sex	0-14		15-44		45-59		60-79		80+	
	Sites	Cases	Sites	Cases	Sites	Cases	Sites	Cases	Sites	Cases
Male										
	All sites	12.37	All sites	151.89	All sites	574.97	All sites	1224.15	All sites	270.95
	Leukemia	4.77	Liver	29.07	Lung	123.07	Lung	338.36	Lung	74.26
	Brain	2.15	Thyroid	22.92	Liver	107.78	Stomach	171.83	Stomach	33.80
	Lymphoma	1.04	Lung	13.98	Stomach	61.97	Colorectum	133.99	Colorectum	31.41
	Bone	0.61	Colorectum	12.18	Colorectum	60.90	Liver	129.04	Liver	22.55
	Kidney	0.43	Leukemia	9.81	Esophagus	40.78	Esophagus	119.27	Prostate	22.31
Female										
	All sites	8.98	All sites	252.79	All sites	597.95	All sites	763.47	All sites	206.44
	Leukemia	3.47	Breast	63.14	Breast	145.54	Lung	151.02	Lung	49.42
	Brain	1.51	Thyroid	60.60	Thyroid	66.79	Colorectum	90.17	Colorectum	28.88
	Lymphoma	0.49	Cervix	26.98	Lung	65.26	Breast	87.75	Stomach	21.68
	Bone	0.46	Lung	12.60	Cervix	59.36	Stomach	63.61	Liver	17.75
	Ovary	0.29	Ovary	10.22	Colorectum	40.21	Liver	53.40	Esophagus	15.61

The estimated overall number of cancer deaths, and the numbers of deaths by age group, sex and cancer type were shown in Table 6. The highest number of cancer deaths occurred in the age group of 60-79 in both men and women. Leukemia, cancer of the brain, lymphoma, and cancers of the liver and bone were the top five deadly cancers for both boys and girls. Liver cancer was the first leading cause of death in men aged 15-59. Lung cancer ranked as the most common cancer for the population of 60 years and above. In women, breast cancer contributed to the most cancer deaths in the age group of 15–44 years, whereas lung cancer took most of the cases of cancer patients aged 45 years or above.

**Table 6:** Estimated cancer deaths of all cancers and five leading cancer types by age and sex in China, 2016 (thousands).

Sex	0-14		15-44		45-59		60-79		80+	
	Sites	Cases	Sites	Cases	Sites	Cases	Sites	Cases	Sites	Cases
Male										
	All sites	5.20	All sites	64.93	All sites	328.64	All sites	857.48	All sites	274.45
	Leukemia	1.95	Liver	22.18	Liver	87.99	Lung	279.49	Lung	82.26
	Brain	1.29	Lung	9.00	Lung	83.85	Stomach	123.31	Stomach	38.34
	Lymphoma	0.34	Leukemia	5.45	Stomach	34.04	Liver	115.00	Colon-rectum	28.41
	Liver	0.28	Stomach	4.45	Esophagus	26.25	Esophagus	90.56	Esophagus	24.24
	Bone	0.21	Brain	4.18	Colon-rectum	19.78	Colon-rectum	62.20	Liver	24.20
Female										
	All sites	3.43	All sites	49.90	All sites	181.93	All sites	440.30	All sites	207.28
	Leukemia	1.22	Breast	8.32	Lung	33.65	Lung	109.03	Lung	53.81
	Brain	0.84	Lung	5.75	Breast	27.37	Liver	46.25	Colorectum	25.66
	Lymphoma	0.19	Cervix	4.63	Liver	17.36	Stomach	45.39	Stomach	24.25
	Liver	0.15	Stomach	4.40	Cervix	15.37	Colon-rectum	39.01	Liver	18.95
	Bone	0.15	Liver	4.06	Stomach	14.29	Esophagus	30.47	Esophagus	16.61

### 3.5 Trends in cancer incidence and mortality

**Fig. 2:**
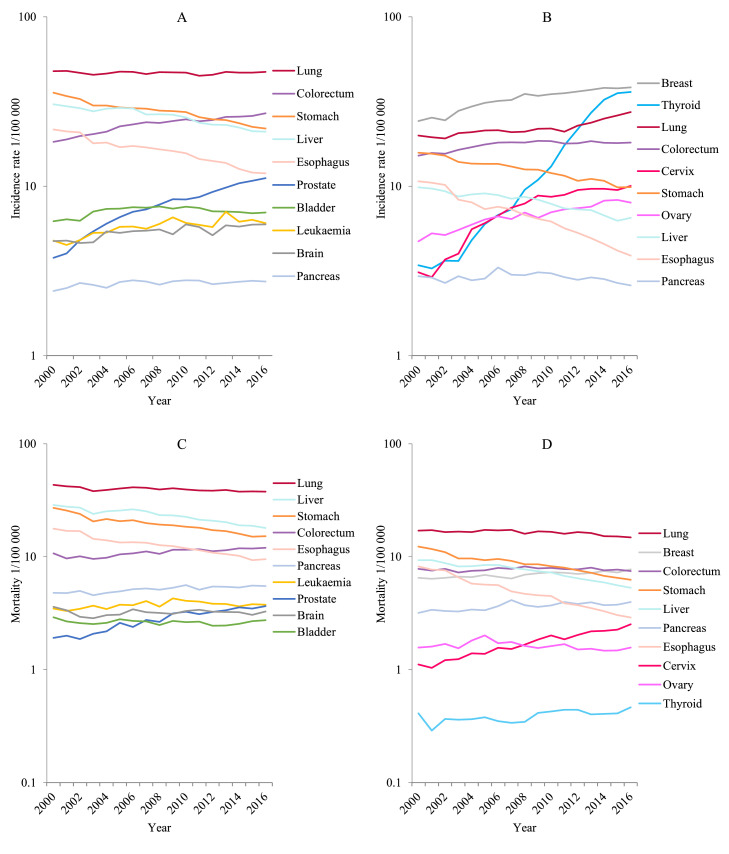
Trends in incidence and mortality rates for selected cancers by sex in China, 2000 to 2016. (A) Male incidence. (B) Female incidence. (C) Male mortality. (D) Female mortality.

Fig. 2 and Table 7 showed the trends of incidence for all cancers combined and selected cancers by sex. The age-standardized incidence rates remained stable for all cancers combined during 2000-2016 in men, but significantly increased by 2.3% per year in women. In men, the average annual percentage change (AAPC) for incidence rates showed an increasing trend for cancers of the prostate (7.1%), colon-rectum (2.4%), leukemia (1.9%), brain (1.5%), pancreas (1%) and bladder (0.8%); whereas the incidence decreased for cancers of the esophagus (3.9%), stomach (3.0%) and liver (2.2%). The trend for lung cancer was stable during 2000 to 2016. In women, the age-standardized incidence rates showed a significant increase for cancers of the thyroid (17.7%), cervix (8.5%), uterus (3.5%), colon-rectum (1.2%), lung (2.1%), and breast (3.0%), but with a decreasing trend for cancers of the esophagus(6.4%), stomach(2.9%) and liver cancer(2.7%).

**Table 7:** Trends in incidence rates for selected cancers by sex in China, 2000 to 2016.

Sex	Site	Trend 1		Trend 2		Trend 3		AAPC		
		Years	APC (95% CI)	Years	APC (95% CI)	Years	APC (95% CI)	2000-2016	2007-2016	2012-2016
**Male**										
	All sites	2000-2016	0.1 (-0.1∼0.3)	—	—	—	—	0.1 (-0.1∼0.3)	0.1 (-0.1∼0.3)	0.1 (-0.1∼0.3)
	Esophagus	2000-2004	-5.3*(-7.9∼-2.5)	2004-2009	-1.8 (-4.6∼1.0)	2009-2016	-4.5*(-5.7∼-3.4)	-3.9*(-4.9∼-2.8)	-3.9*(-4.9∼-3.0)	-4.5*(-5.7∼-3.4)
	Stomach	2000-2004	-4.6*(-6.0∼-3.3)	2004-2009	-1.1 (-2.5∼0.3)	2009-2016	-3.4*(-4.0∼-2.8)	-3.0*(-3.5∼-2.5)	-2.9*(-3.4∼-2.4)	-3.4*(-4.0∼-2.8)
	Colon-rectum	2000-2006	4.2*(3.3∼5.0)	2006-2016	1.3*(1.0∼1.7)	—	—	2.4*(2.0∼2.7)	1.3*(1.0∼1.7)	1.3*(1.0∼1.7)
	Liver	2000-2006	-0.9 (-2.0∼0.3)	2006-2016	-3.0*(-3.5∼-2.5)	—	—	-2.2*(-2.7∼-1.7)	-3.0*(-3.5∼-2.5)	-3.0*(-3.5∼-2.5)
	Pancreas	2000-2016	1.0*(0.7∼1.2)	—	—	—	—	1.0*(0.7∼1.2)	1.0*(0.7∼1.2)	1.0*(0.7∼1.2)
	Lung	2000-2016	-0.1 (-0.2∼0.1)	—	—	—	—	-0.1 (-0.2∼0.1)	-0.1 (-0.2∼0.1)	-0.1 (-0.2∼0.1)
	Prostate	2000-2005	12.5*(10.8∼14.2)	2005-2016	4.8*(4.3∼5.3)	—	—	7.1*(6.6∼7.7)	4.8*(4.3∼5.3)	4.8*(4.3∼5.3)
	Bladder	2000-2006	3.8*(2.5∼5.1)	2006-2016	-1.0*(-1.6∼-0.4)	—	—	0.8*(0.2∼1.3)	-1.0*(-1.6∼-0.4)	-1.0*(-1.6∼-0.4)
	Brain, CNS	2000-2016	1.5*(0.9∼2.0)	—	—	—	—	1.5*(0.9∼2.0)	1.5*(0.9∼2.0)	1.5*(0.9∼2.0)
	Leukemia	2000-2009	3.4*(1.8∼5.0)	2009-2016	-0.1 (-2.3∼2.2)	—	—	1.9*(0.6∼3.1)	0.7 (-0.9∼2.3)	-0.1 (-2.3∼2.2)
**Female**										
	All sites	2000-2016	2.3*(2.1∼2.5)	—	—	—	—	2.3*(2.1∼2.5)	2.3*(2.1∼2.5)	2.3*(2.1∼2.5)
	Esophagus	2000-2004	-7.9*(-11.4∼-4.2)	2004-2010	-4.2*(-6.8∼-1.5)	2010-2016	-7.5*(-9.4∼-5.5)	-6.4*(-7.7∼-5.0)	-6.4*(-7.8∼-5.0)	-7.5*(-9.4∼-5.5)
	Stomach	2000-2016	-2.9*(-3.1∼-2.6)	—	—	—	—	-2.9*(-3.1∼-2.6)	-2.9*(-3.1∼-2.6)	-2.9*(-3.1∼-2.6)
	Colon-rectum	2000-2006	3.3*(2.5∼4.0)	2006-2016	-0.0 (-0.4∼0.3)	—	—	1.2*(0.9∼1.5)	-0.0 (-0.4∼0.3)	-0.0 (-0.4∼0.3)
	Liver	2000-2008	-1.6*(-2.5∼-0.7)	2008-2016	-3.7*(-4.6∼-2.8)	—	—	-2.7*(-3.2∼-2.1)	-3.4*(-4.2∼-2.7)	-3.7*(-4.6∼-2.8)
	Lung	2000-2011	0.9*(0.5∼1.4)	2011-2016	4.6*(3.1∼6.1)	—	—	2.1*(1.6∼2.6)	3.0*(2.2∼3.7)	4.6*(3.1∼6.1)
	Breast	2000-2008	4.6*(3.7∼5.5)	2008-2016	1.4*(0.5∼2.3)	—	—	3.0*(2.4∼3.6)	1.7*(1.0∼2.4)	1.4*(0.5∼2.3)
	Cervix	2000-2007	16.0*(13.0∼19.1)	2007-2016	2.9*(1.1∼4.8)	—	—	8.5*(7.0∼10.0)	2.9*(1.1∼4.8)	2.9*(1.1∼4.8)
	Uterus	2000-2005	5.5*(3.1∼8.1)	2005-2016	2.6*(1.8∼3.3)	—	—	3.5*(2.7∼4.3)	2.6*(1.8∼3.3)	2.6*(1.8∼3.3)
	Brain, CNS	2000-2016	2.9*(2.2∼3.6)	—	—	—	—	2.9*(2.2∼3.6)	2.9*(2.2∼3.6)	2.9*(2.2∼3.6)
	Thyroid	2000-2004	9.5*(1.1∼18.7)	2004-2016	20.6*(18.8∼22.4)	—	—	17.7*(15.3∼20.2)	20.6*(18.8∼22.4)	20.6*(18.8∼22.4)

* The APC is significantly different from zero (*P* < 0.05).

Abbreviations: APC, annual percentage change; CNS, central nervous system; ICD-10, International Classification of Diseases 10^th^ rNevision.

The overall cancer mortality rate presented an annual decrease of 1.2% on average in men, which was mainly resulted from the decreasing esophageal cancer (4.1%), stomach cancer (3.4%), liver cancer (2.7%) and lung cancer (0.6%) (Table 8). However, the rates for prostate cancer (4.6%), colorectal cancer (1.3%) and pancreatic cancer (1.0%) increased during this period. The rates of other cancers such as bladder cancer, brain cancer and leukemia remained stable. In women, there was an upward trend of mortality rates of cancers of the cervix, thyroid and breast but a downward trend of the esophagus, stomach, liver and lung.

**Table 8:** Trends in mortality rates for selected cancers by sex in China, 2000 to 2016.

Sex	Site	Trend 1		Trend 2		Trend 3		AAPC		
		Years	APC (95% CI)	Years	APC (95% CI)	Years	APC (95% CI)	2000-2016	2007-2016	2012-2016
**Men**										
	All sites	2000-2016	-1.2*(-1.4∼-0.9)	—	—	—	—	-1.2*(-1.4∼-0.9)	-1.2*(-1.4∼-0.9)	-1.2*(-1.4∼-0.9)
	Esophagus	2000-2004	-6.2*(-9.0∼-3.4)	2004-2009	-2.4 (-5.2∼0.6)	2009-2016	-4.0*(-5.2∼-2.8)	-4.1*(-5.2∼-3.0)	-3.7*(-4.7∼-2.7)	-4.0*(-5.2∼-2.8)
	Stomach	2000-2004	-5.3*(-7.4∼-3.2)	2004-2016	-2.8*(-3.2∼-2.4)	—	—	-3.4*(-4.0∼-2.9)	-2.8*(-3.2∼-2.4)	-2.8*(-3.2∼-2.4)
	Colon-rectum	2000-2016	1.3*(0.9∼1.7)	—	—	—	—	1.3*(0.9∼1.7)	1.3*(0.9∼1.7)	1.3*(0.9∼1.7)
	Liver	2000-2016	-2.7*(-3.1∼-2.3)	—	—	—	—	-2.7*(-3.1∼-2.3)	-2.7*(-3.1∼-2.3)	-2.7*(-3.1∼-2.3)
	Pancreas	2000-2016	1.0*(0.7∼1.4)	—	—	—	—	1.0*(0.7∼1.4)	1.0*(0.7∼1.4)	1.0*(0.7∼1.4)
	Lung	2000-2016	-0.6*(-0.9∼-0.3)	—	—	—	—	-0.6*(-0.9∼-0.3)	-0.6*(-0.9∼-0.3)	-0.6*(-0.9∼-0.3)
	Prostate	2000-2016	4.6*(3.9∼5.2)	—	—	—	—	4.6*(3.9∼5.2)	4.6*(3.9∼5.2)	4.6*(3.9∼5.2)
	Bladder	2000-2016	-0.3 (-0.8∼0.2)	—	—	—	—	-0.3 (-0.8∼0.2)	-0.3 (-0.8∼0.2)	-0.3 (-0.8∼0.2)
	Brain, CNS	2000-2016	0.1 (-0.6∼0.7)	—	—	—	—	0.1 (-0.6∼0.7)	0.1 (-0.6∼0.7)	0.1 (-0.6∼0.7)
	Leukemia	2000-2009	2.1*(1.0∼3.3)	2009-2016	-1.3 (-2.9∼0.4)	—	—	0.6 (-0.2∼1.5)	-0.5 (-1.7∼0.7)	-1.3 (-2.9∼0.4)
**Women**										
	All sites	2000-2004	-1.7*(-2.7∼-0.8)	2004-2009	-0.2 (-1.2∼0.8)	2009-2016	-4.0*(-5.2∼-2.8)	-1.2*(-1.5∼-0.8)	-1.2*(-1.6∼-0.9)	-1.5*(-1.9∼-1.1)
	Esophagus	2000-2016	-6.3*(-6.6∼-5.9)	—	—	—	—	-6.3*(-6.6∼-5.9)	-6.3*(-6.6∼-5.9)	-6.3*(-6.6∼-5.9)
	Stomach	2000-2004	-6.2*(-8.2∼-4.0)	2004-2009	-1.9 (-4.1∼0.3)	2009-2016	-1.0*(-1.9∼-0.0)	-4.2*(-5.0∼-3.4)	-4.0*(-4.8∼-3.3)	-4.6*(-5.5∼-3.7)
	Colorectum	2000-2004	-0.9 (-3.1∼1.4)	2004-2009	1.5 (-0.8∼3.9)	—	—	-0.2 (-1.0∼0.7)	-0.4 (-1.2∼0.4)	-1.0*(-1.9∼-0.0)
	Liver	2000-2008	-2.0*(-2.8∼-1.2)	2008-2016	-4.7*(-5.5∼-3.9)	—	—	-3.3*(-3.9∼-2.8)	-4.4*(-5.0∼-3.7)	-4.7*(-5.5∼-3.9)
	Lung	2000-2012	-0.3 (-0.8∼0.1)	2012-2016	-2.7*(-5.0∼-0.3)	—	—	-0.9*(-1.5∼-0.3)	-1.4*(-2.3∼-0.4)	-2.7*(-5.0∼-0.3)
	Breast	2000-2016	1.0*(0.8∼1.3)	—	—	—	—	1.0*(0.8∼1.3)	1.0*(0.8∼1.3)	1.0*(0.8∼1.3)
	Cervix	2000-2016	5.4*(4.9∼5.9)	—	—	—	—	5.4*(4.9∼5.9)	5.4*(4.9∼5.9)	5.4*(4.9∼5.9)
	Uterus	2000-2005	3.0 (-0.1∼6.3)	2005-2016	-1.9*(-2.8∼-1.0)	—	—	-0.4 (-1.4∼0.7)	-1.9*(-2.8∼-1.0)	-1.9*(-2.8∼-1.0)
	Brain,CNS	2000-2016	-0.4 (-1.0∼0.2)	—	—	—	—	-0.4 (-1.0∼0.2)	-0.4 (-1.0∼0.2)	-0.4 (-1.0∼0.2)
	Thyroid	2000-2016	1.6*(0.6∼2.6)	—	—	—	—	1.6*(0.6∼2.6)	1.6*(0.6∼2.6)	1.6*(0.6∼2.6)

* The APC is significantly different from zero (*P* < 0.05).

Abbreviations: APC, annual percentage change; CNS, central nervous system; ICD-10, International Classification of Diseases 10^th^ revision.

## Discussion

Cancer is a major public health problem in China. In this study, we analyzed the burden of cancer in China in 2016 using data from 487 qualified cancer registries. We estimated that about 4,064,000 new cancer cases and 2,413,500 cancer deaths in China in 2016. Cancer incidence in urban areas was higher than that in rural areas. Lung cancer was the most common cancer in China as well as the first leading cause of cancer deaths. Age-standardized incidence rates stayed stable in men but increased by 2.3% per year in women during 2000-2016. Age-standardized mortality rates decreased by 1.2% per year both in men and women. The updated statistics for cancer incidence and mortality overall and by cancer type in China may provide scientific evidence for policymakers, researchers, and clinicians.

The results of this study were the estimation for cancer incidence and mortality in China in 2016. For all cancers combined, the numbers of new cases and deaths in China estimated in this study were relatively low compared with the estimation given by Globocan2020^6^, the latest global cancer prediction from IARC, but closer to the results of Globocan 2018^7^. However, the patterns for specific cancer types were quite different from the Globocan database (**Supplementary Table 1 and 2**). The methods used to estimate the global cancer incidence and mortality in 2020 were based on the most recent data supplied by population-based cancer registries (PBCR) of IARC for Cancer Incidence in Five Continents (CI5) Vol. XI (data from 2008 to 2012) ^8^^,^^9^. Nevertheless, cancer registration work in China in recent years has made great progress and development. With the implementation of Chinese Cancer Registration Management Regulation^10^, preparation and promotion of the standardization of cancer registration work, the population covered by registration gradually has expanded and the quality of registration data has been steadily improved^2^. Moreover, China has established more than 1,600 cancer registries, covering more than 890 million people by the beginning of 2022. The results of this study should be more representative of the actual cancer burden in China compared with the results of Globocan estimates.

The numbers of overall new cases and deaths of cancer in China in 2016 was higher than previous years^11^^,^^12^. With the social and economic development in China, the life expectancy has increased and the population structure is aging. Considering aging is an established risk factor for cancer, the increasing cancer burden of China may be partly due to expanding population during the past decades. Tobacco consumption is one of the main risk factors for many cancer types including lung cancer, esophageal cancer, stomach cancer, etc. China has the world's largest smoking population, with an estimated 350 million smokers and 740 million passive smokers^13^. Previous studies have shown that smoking accounts for more than 20% of cancer deaths in China^14^, ^15^, ^16^. Effective tobacco control has been shown to be an effective intervention method to reduce cancer incidence in western countries^17^. In comparison, only some cities or regions in China such as Beijing and Shanghai have implemented a ban on smoking in indoor public places. Nationwide intervention in smoking control is urgently needed and the focus should be put on the prevention of smoking among women. Although smoking is one of the major risk factors for lung cancer, we should also pay more attention to the continuous increase in lung cancer incidence rate among non-smoking female population, especially in rural areas. The incidence rate of lung cancer increased from 2000 to 2016, and the average annual percent change reached 2.1% for women, and even increased by 4.6% per year in the last 5 years. This may be related to indoor cooking and the air pollution exposure.

Similar to the global increases of colorectal cancer and breast cancer, increasing trends of these cancers were also observed in the Chinese population. The obesity prevalence in China rose from 3.1% to 8.1% from 2004 to 2018^18^. Given that the proportion of population with obesity and physical inactivity in China is still increasing, these modifiable risk factors may play a role in the increase of colorectal cancer and breast cancer. Therefore, modifiable risk factors including unhealthy lifestyle, obesity, physical inactivity, and other risk factors contributed over 40% of the cancer incidence and mortality in China^19^^,^^20^, so the healthy lifestyle promotion is needed for effective cancer control in the country. Thyroid cancer experienced the largest increase in incidence among all cancer types, whereas its mortality remained stable, indicating overdiagnosis may play a part with a rapid transition to a higher socioeconomic level of the country's economy^21^. On the bright side, our study showed that esophageal cancer, stomach cancer and liver cancer showed a continuous decreasing trend for age-standardized incidence and mortality rates. The decreasing trends of liver cancer may be attributed to decreased consumption of aflatoxins-contaminated food, improved quality of water, as well as the Hepatitis B virus vaccination. Specially, neonatal Hepatitis B virus vaccination has been made free for all children since 2002, and the vaccination rates reached 99.6% in 2015^22^. Endoscopic screening has been shown to be an effective intervention method to reduce esophageal cancer incidence and mortality^23^^,^^24^. And we observed a more favorable trend toward early-stage diagnosis in the Chinese population especially in the areas with systematic esophageal cancer control programs including primary cancer prevention and cancer screening^25^. The decreasing trends of upper gastrointestinal cancers in China may further support the pivotal role of endoscopic cancer screening in high-risk areas.

There are several strengths for this analysis. First, this is a systematic use of the most updated and representative data of China, including 487 cancer registries, covering about 381,565,422 population, which accounted for 27.60 % of the national population by the end of 2016. In particular, this study reported the cancer incidence, mortality and temporal trend in China by sex, age group and region. Detailed information from different perspectives can be provided for cancer prevention and control.

There are also some limitations in this study. First, the estimations in this analysis relied upon the best available registry data to calculate the incidence and mortality at the country level. Secondly, there remains a lack of sufficient high-quality data in some areas such as Xinjiang, Tibet, and Qinghai province. However, this estimation was stratified by urban and rural areas, so the poor quality of some provincial data does not affect the national estimation results. Finally, the data used in the trend analysis section only covered 22 cancer registries, representing a relatively small population and not fully reflecting the overall trend changes in China.

In conclusion, the burden of cancer in China is heavy and is expected to continue increasing in the next decade. China has issued a series of health policies to prioritize the promotion of cancer control, and the State Council has established the inter-ministerial joint conference system to prevent and control major chronic diseases. This study showed that the burden of cancer in China continued to increase mainly due to the aging of the population, but after adjusting the age structure of the population, the trend changes are different for each cancer site. Some showed an upward trend, some declined or remained stable, but the digestive system cancers showed a downward trend regardless of the incidence and mortality, indicating that the effect of prevention and control measures in China have now taken effect. Specially, a significant decline in cancer mortality has been observed for the first time in this study. Not only should the cancer prevention and control continue maintaining existing strategies such as targeted prevention and early detection, and treatment programs be carried out to control the increasing cancer burden, but also investments should be increased in clinical treatment and basic research of cancer to accelerate progress against cancer and improve cancer survival in China.

## Declaration of competing interest

The authors declare that they have no conflict of interests.

## Appendix Supplementary materials

Image, application 1
