# Supplementary material for: Cancer incidence and mortality in China, 2016
Source: J Natl Cancer Cent. 2022 Feb 27;2(1):1–9. doi: 10.1016/j.jncc.2022.02.002 (PMC11256658; doi:10.1016/j.jncc.2022.02.002)
Supplement: Supplementary file 1 [file mmc1.pdf]

Supplementary materials

Cancer incidence and mortality in China, 2016

Rongshou Zheng, Siwei Zhang, Hongmei Zeng, Shaoming Wang, Kexin Sun, Ru Chen, Li Li, Wenqiang Wei, Jie He

Supplementary Table 1

Comparation of the estimated cancer incidence in China in 2016 with Globocan.

ICD10	Sites	China estimate 2016			GLOBOCAN2018			GLOBOCAN2020		
		Cases	Crude rate (1/100 000)	ASR (1/100 000)	Cases	Crude rate (1/100 000)	ASR (1/100 000)	Cases	Crude rate (1/100 000)	ASR (1/100 000)
ALL	All sites	4064000	293.91	186.46	4285033	301.10	201.70	4568754	317.42	206.35
C11	Nasopharynx	52000	3.76	2.51	60558	4.30	3.00	62444	4.34	3.01
C15	Esophagus	252500	18.26	11.13	307359	21.60	13.90	324422	22.54	13.92
C16	Stomach	396500	28.68	17.59	456124	32.10	20.70	478508	33.25	20.74
C18-21	Colorectum	408000	29.51	18.05	521490	36.60	23.70	555477	38.59	24.07
C22	Liver	388800	28.12	17.65	392868	27.60	18.30	410038	28.49	18.35
C25	Pancreas	100400	7.26	4.36	116291	8.20	5.20	124994	8.68	5.30
C32	Larynx	25700	1.86	1.17	27832	2.00	1.30	29135	2.02	1.27
C33-34	Lung	828100	59.89	36.46	774323	54.40	35.10	815563	56.66	35.08
C50	Female breast	306000	45.37	29.05	367900	53.30	36.10	416371	59.39	39.38
C53	Cervix	119300	17.69	11.34	106430	15.40	10.70	109741	15.65	10.78
C56	Ovary	57200	8.47	5.59	52971	7.70	5.30	55342	7.89	5.35
C61	Prostate	78300	11.05	6.72	99322	13.60	9.10	115426	15.64	10.34
C67	Bladder	82300	5.95	3.53	82270	5.80	3.70	85694	5.95	3.67

C70-72	Brain, CNS	109000	7.88	5.57	76494	5.40	4.10	79575	5.53	4.08
C73	Thyroid	202600	14.65	10.37	194232	13.60	10.10	221093	15.36	11.41
C91-95	Leukemia	85800	6.21	5.1	82607	5.80	5.10	85404	5.93	5.15

Abbreviations: ASR, age-standardized rate; CNS, central nervous system; ICD10, International Statistical Classification of Diseases 10th Revision.

Supplementary Table 2

Comparation of the estimated cancer mortality in China in 2016 with Globocan.

ICD10	Sites	China estimate 2016			GLOBOCAN2018			GLOBOCAN2020		
		Deaths	Crude rate (1/100 000)	ASR (1/100 000)	Deaths	Crude rate (1/100 000)	ASR (1/100 000)	Deaths	Crude rate (1/100 000)	ASR (1/100 000)
ALL	All sites	2413500	174.55	105.19	2865174	201.30	130.10	3002899	208.63	130.47
C11	Nasopharynx	26700	1.93	1.24	31413	2.20	1.50	34810	2.42	1.59
C15	Esophagus	193900	14.02	8.28	283433	19.90	12.70	301135	20.92	12.78
C16	Stomach	288500	20.87	12.3	390182	27.40	17.50	373789	25.97	15.99
C18-21	Colon-rectum	195600	14.14	8.13	247563	17.40	10.90	286162	19.88	12.07
C22	Liver	336400	24.33	15.07	368960	25.90	17.10	391152	27.18	17.38
C25	Pancreas	87900	6.35	3.75	110390	7.80	4.90	121853	8.47	5.15
C32	Larynx	14300	1.03	0.61	15698	1.10	0.70	15814	1.10	0.68
C33-34	Lung	657000	47.51	28.09	690567	48.50	30.90	714699	49.66	30.49
C50	Female breast	71700	10.62	6.39	97972	14.20	8.80	117174	16.71	10.03
C53	Cervix	37200	5.52	3.36	47739	6.90	4.40	59060	8.42	5.31
C56	Ovary	27200	4.04	2.45	30886	4.50	2.90	37519	5.35	3.33
C61	Prostate	33600	4.75	2.73	51895	7.10	4.70	51094	6.92	4.69
C67	Bladder	33700	2.44	1.31	38208	2.70	1.60	39393	2.74	1.62
C70-72	Brain, CNS	58500	4.23	2.91	63860	4.50	3.20	65204	4.53	3.18
C73	Thyroid	8300	0.6	0.37	8603	0.60	0.39	9261	0.64	0.40
C91-95	Leukemia	55700	4.03	2.98	65531	4.60	3.50	61694	4.29	3.28

Abbreviations: ASR, age-standardized rate; CNS, central nervous system; ICD10, International Statistical Classification of Diseases 10th Revision.
